# Evaluation of pathology resources for cervical cancer detection between 2018 and 2022: a retrospective study at Moi Teaching and Referral Hospital, Western Kenya

**DOI:** 10.1186/s12885-025-13563-9

**Published:** 2025-02-05

**Authors:** Nelson Anangwe, Jon Steimgrimson, Susan Cu-Uvin

**Affiliations:** 1 School of Public Health, Moi University, Eldoret, 4606 - 30100 Kenya; 2 School of Public Health, Brown University, 121 S Main St, Providence, RI 02903 USA

**Keywords:** Histopathology laboratory, Cervical cancer, Diagnosis, LMICs, Histo-technician, Pathologist, Kenya

## Abstract

**Background:**

Cervical cancer cases are increasing in sub-Saharan Africa, particularly in Kenya, exacerbated by inadequate histopathology resources, posing a significant barrier to timely diagnosis and treatment. There has been little research on the availability and evolution of histopathology resources for diagnosing cervical cancer over the years. This retrospective study evaluated this evolution at Moi Teaching and Referral Hospital in Kenya between 2018 and 2022.

**Methods:**

We used a mixed-methods approach. An in-depth interview was conducted with one of MTRH’s pathology laboratory staff to assess the equipment, personnel, and quality control trends between 2018 and 2022. A thematic analysis was conducted in NVivo. We also retrospectively conducted a comprehensive inventory review of laboratory resources from 2018 to 2022 via purposive sampling. Microsoft Excel and Stata version 17 were utilized for descriptive statistical analysis. Turnaround time (TAT) was assessed against the UK’s National Health Service Cervical Screening Program guidelines.

**Results:**

The number of histopathology laboratory personnel at MTRH increased from 2018 to 2022, during which the facility included two pathologists, one records person, and one office administrator. Cervical cancer biopsy samples processed by the histopathology lab increased from 225 in 2018 to 674 in 2022. However, the histopathology personnel-to-population ratio decreased from 1.5 pathologists and 2.7 histo-technicians per 100,000 in 2018 to 1.4 pathologists and 1.8 histo-technicians per 100,000 in 2022. Despite this decrease, lab equipment, automatic tissue processors and embedding machines were added, and an average 14-day turnaround time was maintained for cervical cancer pathology reports.

**Conclusions:**

Our study highlights a growing burden of cervical cancer with biopsy samples processed by the MTRH histopathology laboratory, increasing from 225 in 2018 to 674 in 2022. Despite challenges such as a declining staff-to-patient ratio and limited resources, the lab maintained a commendable 14-day turnaround time, supporting timely cervical cancer diagnoses. These findings emphasize the need for continued investment in pathology resources and personnel to enhance diagnostic capacity and address the rising incidence of cervical cancer in Kenya and similar low-resource settings. The decline in the personnel-to-patient ratio underscores challenges in diagnosis, emphasizing the need to address workforce and infrastructure gaps to improve patient care within similar low-resource settings.

**Supplementary Information:**

The online version contains supplementary material available at 10.1186/s12885-025-13563-9.

## Background

Cervical cancer is a chronic illness resulting from persistent infection of the cervix by HPV types 16 and 18 [1]. In 2020, there were an estimated 604,127 annual cervical cancer cases globally, representing 3.1% of all cancer cases [2]. Approximately 84% of newly reported cases and between 87% and 90% of fatalities are concentrated in low- and middle-income countries (LMICs) [3]. These cases are equivalent to more than one-quarter of a million deaths annually in LMICs due to inadequate human papillomavirus (HPV) vaccination programs, screening, and gross deficiencies in diagnostic infrastructure [2, 4]. In contrast, incidence rates have decreased by more than half in high-income countries (HICs) during the past 30 years due to the integration of structured screening and diagnosis initiatives [4]. By 2030, cervical cancer mortality rates are projected to increase by approximately 25% in most LMICs [5]. 

Histopathology has been the clinical and scientific foundation for cervical cancer diagnosis and treatment because it plays a vital role in determining the extent of the abnormality [6]. Inadequate pathological resources for cervical cancer diagnosis can result in delayed diagnosis and treatment [7]. However, the shortage of skilled technicians and well-equipped laboratories in developing countries has significantly hindered the service [8–10]. As a result, the disease progresses, limiting treatment options and impeding efforts to reduce mortality [7, 11]. Among women with persistent low-grade abnormalities, such as HPV lesions and advanced cervical cancer lesions, histology plays a vital role in determining the extent of the abnormality [6]. However, despite the critical role of pathology in various aspects of cancer care and control, sub-Saharan African countries have, at most, only a tenth of the pathology coverage compared to high-income nations [12]. 

In sub-Saharan Africa (SSA), limited resources are crucial to integrated cervical cancer care [13]. A crucial setback to integrated cervical cancer care in sub-Saharan Africa (SSA) is its limited infrastructure and human capacity [13]. Pathologists play a critical role in diagnostics, with an estimated 95% of clinical pathways relying on them [14, 15]. Despite their role, they are significantly limited in low and middle-income countries, with only about 30% of patients accessing urgent pathology services [16]. World Health Organization (WHO) reports indicate approximately five pathologists per 100,000 people in HICs compared to most SSA countries, reporting approximately one pathologist per 1 million people [17]. For instance, the United States and Canada boast 6.5 and 4.81 pathologists per 100,000, respectively, compared to less than two pathologists per 100,000 in sub-Saharan Africa [14, 15]. Due to limited pathology facilities and skilled human resources in many LMICs, the WHO’s global strategy to accelerate eliminating cervical cancer by 2030 seems to be a pipedream [18, 19]. 

In Kenya, cervical cancer is the second most common type of cancer among women after breast cancer [20, 21]. The quality of cervical cancer pathology infrastructure and trained clinical staff varies across regions, with rural Kenyan health facilities reporting limited access [22]. Approximately 5,236 women in Kenya are diagnosed with cervical cancer, with an estimated 3,211 mortalities occurring annually [23, 24]. Previous studies have indicated that cancerous lesions are often detected at advanced invasive stages of cervical cancer, resulting in a protracted illness upon diagnosis due to a low pathologist-to-patient ratio and skilled laboratory technologists and histo-technicians [8, 25, 26]. Inadequate pathological resources are a significant barrier to timely and accurate diagnosis and treatment of cervical cancer. Timely detection and prompt initiation of treatment are critical for effectively managing cervical cancer cases.

### Specific aims

This study assessed the progression of histopathology resources available for diagnosing cervical cancer within the Moi Teaching and Referral Hospital (MTRH) histopathology department between 2018 and 2022.

### Study justification

The literature on cervical cancer has focused on the factors driving screening uptake and the barriers to accessing treatment in developing countries. However, there is limited research on the evolution of histopathology resources, such as the availability of skilled laboratory personnel and equipment and quality control measures across time. Given that cervical cancer is primarily diagnosed through histopathological examination of tissue samples, the adequacy of these services directly impacts the accuracy, timeliness, and quality of cervical cancer diagnosis and subsequent patient management. Additionally, in regions like sub-Saharan Africa, where cervical cancer incidence is high, the ability to detect and manage the disease relies heavily on functional histopathology services. Therefore, auditing these indices is essential to understand the capacity of the health system to address the cervical cancer burden.

To address this gap, our study aimed to retrospectively evaluate resources at the MTRH histopathology laboratory between 2018 and 2022. This study’s results will contribute to the body of evidence supporting the significance of histopathology resources in reducing the burden of cervical cancer, particularly in resource-limited settings such as Kenya.

## Methods

### Study design and setting

This manuscript is based on primary and secondary data collected from MTRH in Western Kenya using a retrospective mixed method of qualitative and quantitative data. We purposively selected the MTRH due to its specialized gynecological services, higher cervical cancer cases, and long-standing cancer registry. The facility is Kenya’s second largest national hospital, located in Eldoret town, North Rift area of Western Kenya. It has a bed capacity of approximately 1000 beds [27], and serves 22 counties with an approximate population of 25 million [28], representing 47% of the Kenyan population [29]. MTRH, in collaboration with the Academic Model Providing Access to Healthcare (AMPATH), runs extensive community outreach and screening programs aimed at early detection of cervical cancer. These screenings focus on women of reproductive age and emphasize Visual Inspection with Acetic Acid (VIA) and Pap smears as cost-effective methods for early detection. These programs extend into rural communities, ensuring that underserved populations have access to screening.

### Sampling and study population

The histopathology laboratory technologist was purposively selected for the interview, and census sampling was used to inventory all histopathology equipment, quality control measures, and personnel for the specified investigation period. The histopathology department head guided the recruitment of a laboratory technologist based on their expertise, experience, and knowledge of the facility’s histopathology resources over the past ten years.

### Data collection

We informed the department of the study purpose, data utilization, and any effects of data collection on normal hospital operations. Data was collected between October 2023 and February 2024 upon pretesting of the combined histopathology inventory checklist and semi-structured interview guide. The tool was validated by a histopathology technician and a cervical cancer specialist and operationalized in English due to the interviewees’ proficiency. Upon signing an informed consent form, we interviewed the laboratory technician, corroborating the details with the histopathology inventory to capture data for the study period. We also utilized an observation checklist to document resources available in the histopathology laboratory. The guide comprised open-ended questions on three conceptual topics: (1) *trained personnel* were compared to the WHO’s Workload Indicators of Staffing Need (WISN) tool for laboratory staffing with a guideline ratio of more than two pathologists and two histo-technicians per 100,000 people [30]. (2) Quantity and competence of e*quipment*, guided by the International Standards Organization’s standards for medical laboratories, ISO 15189:2012, which recommends at least two pieces of each piece of equipment for critical function for backup purposes [31]. (3) *Quality control* was compared to the WHO’s annual quality control performance for all histopathology laboratories, which varied by workload [32]. We assessed the *turnaround time* (TAT) in line with the United Kingdom National Health Service Cervical Screening Program (NHSCSP) guidelines of 14 days for relaying biopsy tissue reports to patients [33]. 

### Data analysis

**Fig. 1:**
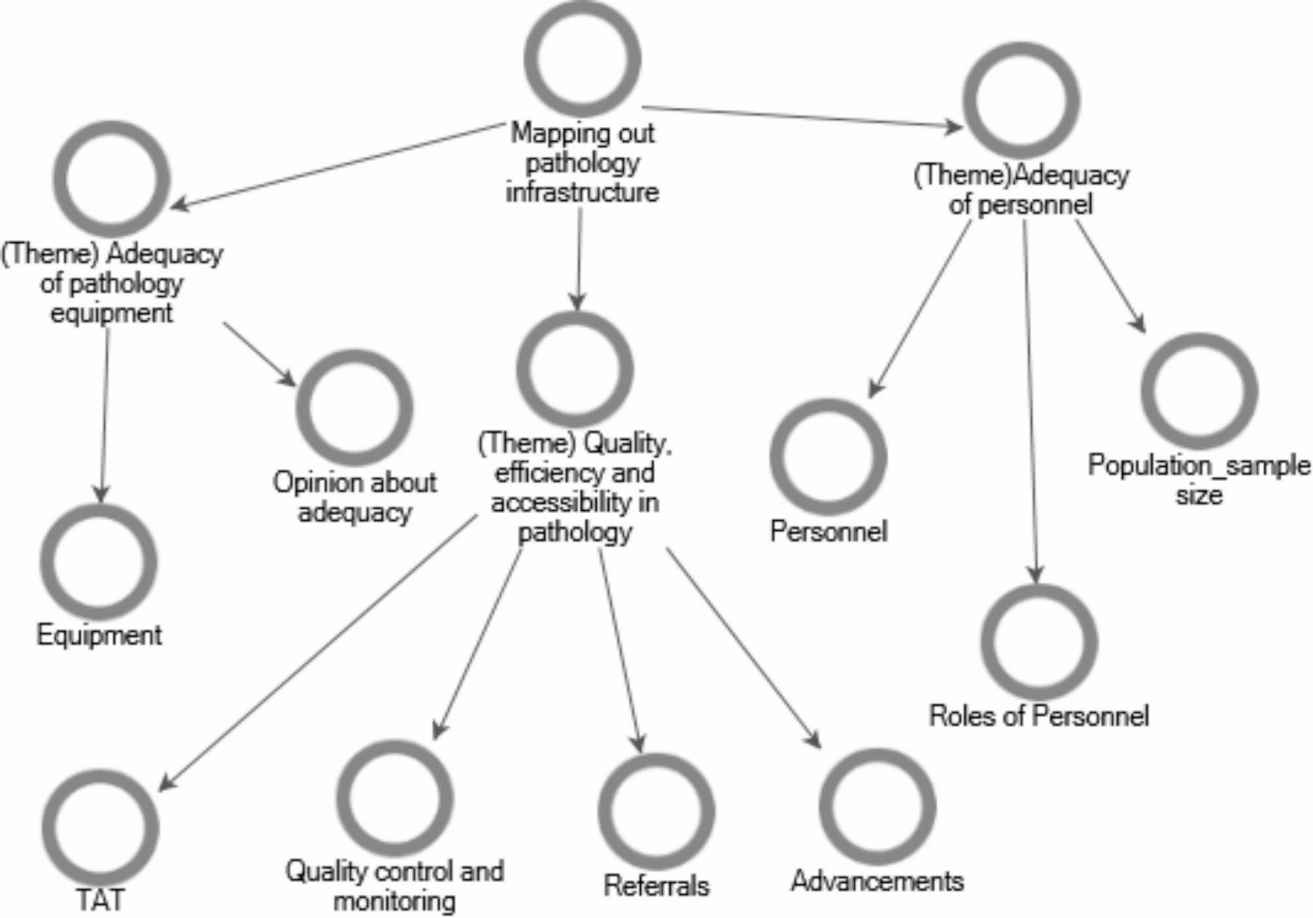
Flow diagram of emerging themes from the interviews and inventory

Initially, we thoroughly reviewed the transcript and documented observations, cross-referencing them with inventory records. Thematic analysis was performed using NVivo version 14. We then revisited the transcript reading and re-reading to familiarize ourselves with the content, noting initial codes and patterns. We employed thematic content analysis to generate critical themes [34]. For this analysis, we imported a priori codes into NVivo and integrated them into emerging codes from the data. Following a process of open coding and identifying relevant segments, the codes were organized into more focused codes and then restructured to create descriptive codes, capturing vital concepts and relationships. This coding process enabled us to refine and revise emerging themes through an iterative process describing trends and outcomes of the findings (Fig. 1) [35].

We then merged and defined each theme grounded in the data to provide a consistent interpretation of the data [36]. Using the observational notes, we corroborated and contrasted the themes from the transcript and the inventory reports to identify patterns across the data. This enhanced the depth and reliability of your analysis.

### Ethical approval

This study was conducted following ethical approval from the MTRH/Moi University Institutional (no. 0004537) and Brown University Institutional Review Boards (no. STUDY00000244). Before collecting data, permission was obtained from the MTRH histopathology laboratory department’s head. Informed consent to participate was obtained from all participants (laboratory technicians) involved in the study. Before data collection, each participant was provided with detailed information about the study’s purpose, procedures, potential risks, and benefits. The participants were assured of their confidentiality and right to withdraw from the study at any time. Written consent was collected from all participants, confirming their voluntary agreement to participate. The Data Protection Act 2019, the Health Act 2017 of Kenya, and the United States data privacy policy were strictly followed during the process.

## Results

### Laboratory staffing

The interview highlighted key details about staffing adequacy and its impact on providing quality services across the years at the MTRH histopathology laboratory. The number of personnel at the histopathology laboratory increased: two pathologists, one records personnel, and one office administrator (Table 1). Consequently, the number of biopsy tissues examined increased from 3000 in 2020 to 7000 in 2022, with approximately 8% and 9.5% being cervical cancer biopsies, respectively.

**Fig. 2:**
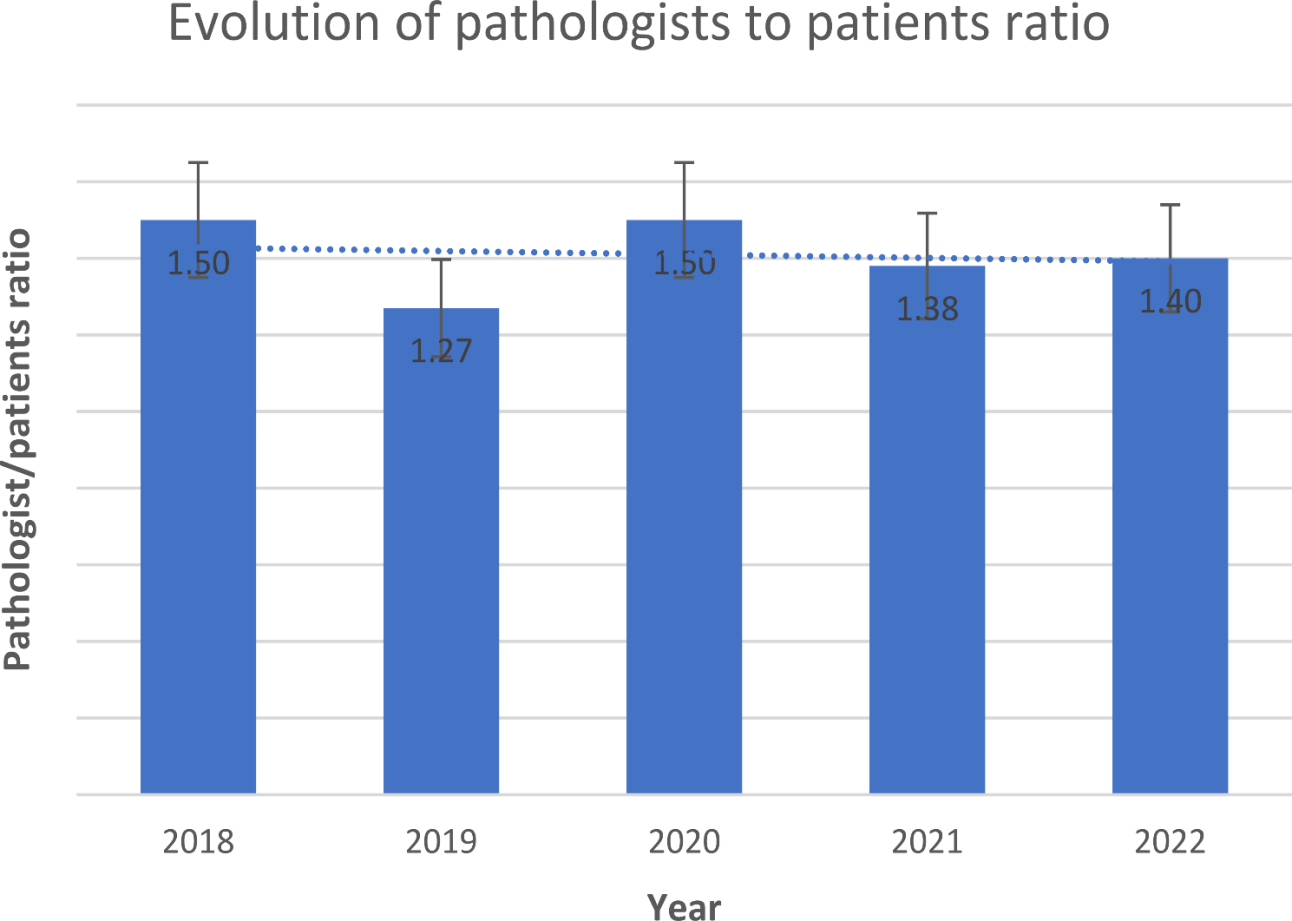
Bar graph showing the evolution of pathologist-patient ratio

**Fig. 3:**
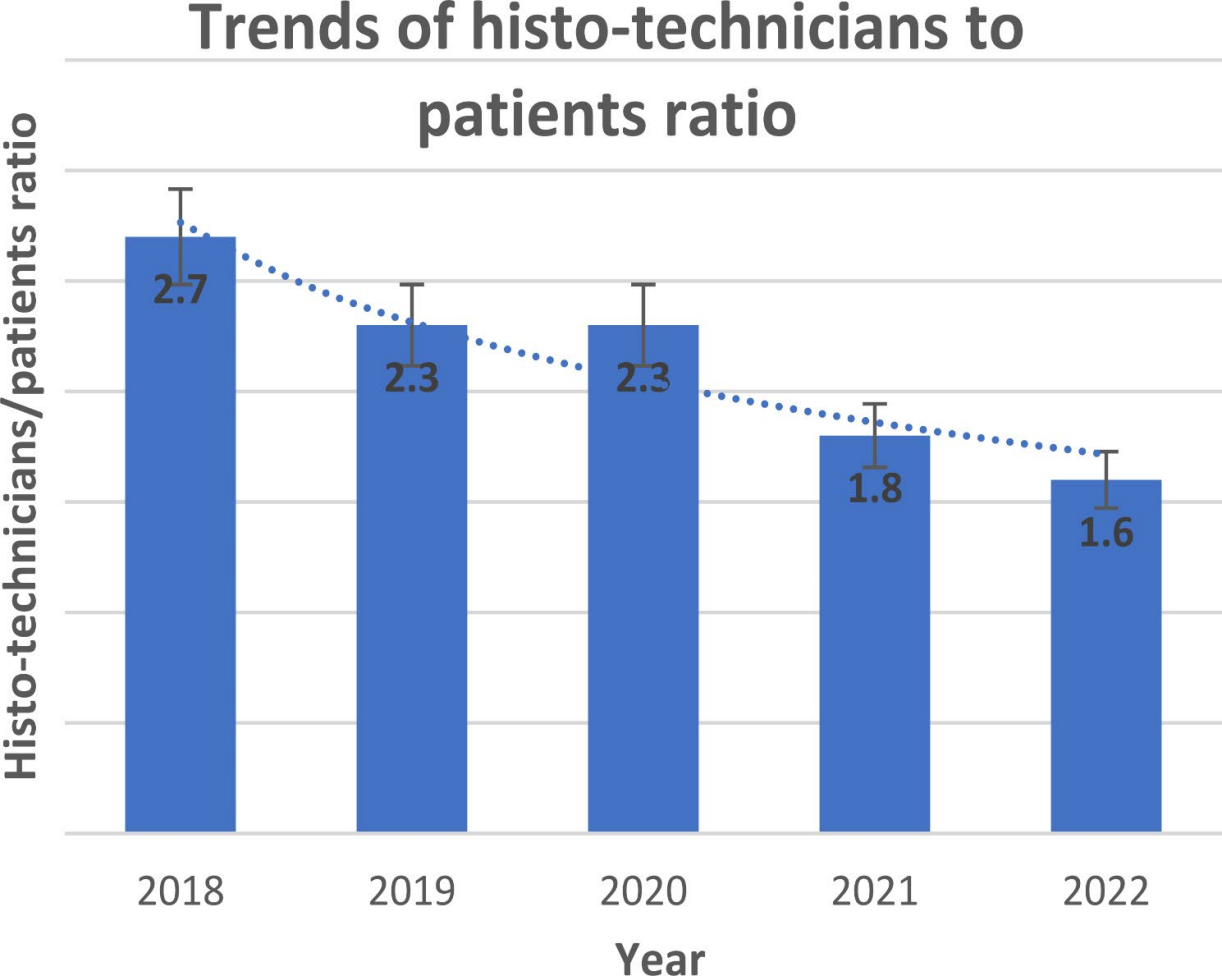
Bar graph and stacked bar chart showing trends in the histo-technicians: patient ratio

The MTRH Records and Information Services Department reported providing care to approximately 350,000 and 500,000 patients annually between 2018 and 2022. Comparing the patient population to the laboratory staff, the histopathology personnel-to-population ratio decreased from 1.5 to 1.4 for pathologists (Figs. 2) and 2.7 to 1.8 for histo-technicians (Fig. 3) per 100,000 in 2018 and 2021, respectively.

**Table 1:** Pathology personnel at the MTRH histopathology laboratory

	2018	2019	2020	2021	2022
Pathologists	5	5	6	7	7
Lab technologists	9	9	9	9	8
Records personnel	0	0	1	1	1
Office Administrators	1	1	2	2	2
Lab assistants	1	1	1	1	1
Total number of specimen	2490	2970	3000	7100	7090
Number of cervical samples	225	215	240	500	674
**MTRH patient census**	**331**,**036**	**393**,**594**	**397**,**399**	**507**,**502**	**493**,**780**

*The pathologist and histotechnician-to-patient ratio was computed by dividing the respective personnel’s number by the total number of inpatients and outpatients each year

### Histopathology equipment

The interview highlighted that all the equipment acquired at the commissioning of the histopathology laboratory in 2012 were manually operated. However, over the years, the facility added laboratory equipment such as an automatic microtone, automatic tissue processor, embedding machine, and immunohistochemistry machine (Table 2).

**Table 2:** Inventory of equipment

	2018	2019	2020	2021	2022
Cytology centrifuge (manual)	1	1	1	1	1
Automatic tissue processor	1	1	1	2	2
Embedding machines	1	1	1	1	2
IHC machine	1	1	1	1	1
Microtones	2	2	1	2	2

However, we noted a diminished capacity in early 2020 when a microtone machine broke down, so we reverted to using the manual machine acquired in 2012 for about a year (Table 2). However, the hospital eventually purchased another in 2021. This likely impeded the efforts of biopsy tissue examination.

…The AMPATH Cervical Cancer Program donated an automatic microtome in 2018, which we used until early 2020 then it malfunctioned due to overload. The hospital eventually replaced them with two new ones in 2021.

The interviewee emphasized the need for automatic *backup equipment* for histopathology examinations, noting that manual equipment were prone to measurement errors when processing high workloads. Additionally, the staining of tissue slides is performed manually using standard histological stains such as hematoxylin and eosin (H&E). This process is routinely employed for diagnostic purposes in cervical cancer and other pathological conditions.

“*Immunohistochemistry… Yes*,* but we like backup machines so that in case of a breakdown*,* we don’t go back to manual types that are prone to many errors. So going forward*,* we would be comfortable when we get automatic backups*.”

### Quality control and measures

The MTRH histopathology laboratory was registered for external quality assessment (EQA) in 2018 and implemented throughout the study period (Table 3). The EQA program permits laboratories to determine the accuracy and precision of their equipment by testing samples with known characteristics such as concentration levels and other physical properties.

**Table 3:** Histopathology laboratory quality control

	2018	2019	2020	2021	2022
Quality checks	EQA Done	EQA Done	EQA Done	EQA Done	EQA Done
Referrals	Mostly lymphomas	Only Upon request	Only Upon request	Only Upon request	Only upon request
Turnaround time	14 days	14 days	14 days	14 days	14 days

Ref: According to the NHSCSP Guidelines, the TAT from the date the sample is taken to when the patient receives the biopsy report within 14 days

The MTRH Department of Biomedical Engineering evaluates all pathology equipment, personnel standards, and laboratory quality annually.

*“…an annual evaluation for all equipment in the laboratory is done by the Department of Biomedical Engineering…. Equipment servicing is performed annually by those given the contract to supply this equipment.” “…Over 80% of biopsy specimen results are obtained in 14 days.”* (Table 3).

## Discussion

Our study revealed a progressive increase in pathology personnel, equipment, and cervical cancer biopsy samples at the Moi Teaching and Referral Hospital histopathology laboratory across the investigated period. These findings offer profound insights and aim to improve cervical cancer diagnostics at MTRH and hospitals in similar settings.

### Evolution of pathology resources at the MTRH histopathology laboratory

Our study demonstrated that between 2018 and 2022, cervical cancer biopsy samples processed by the histopathology lab increased from 225 in 2018 to 674 in 2022. This surge reflects both the increasing demand for cervical cancer screening services and the growing burden of the disease. Despite the increase in demand, the lab experienced a decline in the personnel-to-patient ratio, from 1.5 pathologists and 2.7 histo-technicians per 100,000 in 2018 to 1.4 pathologists and 1.8 histo-technicians in 2022. This reduction highlights a disparity between the growing workload and available personnel, likely impacting the lab’s capacity to handle the rising cases effectively. This finding is consistent with other studies that reported a low pathologist-to-patient ratio in SSA patients [5, 14, 15]. Limited personnel impede the performance of laboratory tasks in many SSA countries [5]. Studies suggest that low personnel ratios, like those observed at MTRH, are insufficient to meet the diagnostic demands of patients in sub-Saharan Africa, where the burden of cervical cancer continues to rise. Abdulkareem et al., [37], reported that most countries in SSA have an average ratio of 0.1 pathologists per 100,000 people, but our study revealed a higher number of approximately 1.4 pathologists per 100,000 people in 2022.

Our results revealed a lower pathologist-patient ratio than most HICs, aligning with other studies that have reported a high 6.7 pathologists per 100,000 people, such as the United States and Canada [17]. Our findings are lower than those from the WHO WISN guideline tool for laboratory staffing, which recommends more than two pathologists and histo-technicians per 100,000 people. This shortage likely challenged bridging the growing demand for histopathology services between 2018 and 2022 [30]. Research indicates that low staff proportion enables only 30% of patients to access urgent pathology services [16, 31]. 

Despite adding histopathology equipment over five years, most of these malfunctioned due to overload. Our study identified a persistent challenge of relying on manual backup equipment, mainly for immunohistochemistry machines and centrifuges. Studies have shown that dependence of histopathology on manually operated equipment can lead to measurement errors, resulting in increased turnaround time and decreased efficiency in processing specimens [38, 39]. These shortages place the MTRH histopathology laboratory below the required medical laboratories’ standards of the International Standards Organization (ISO 15189:2012) benchmark [31]. While these findings are consistent with studies performed in 30 sub-Saharan African countries in 2016 reporting a shortage of laboratory equipment, the histopathology lab maintained a 14-day turnaround time (TAT), meeting international standards [40]. This TAT period and the implementation of annual evaluation quality control measures closely align with the UK National Health Service Cervical Screening Program (NHSCSP) guidelines of 10–14 days [33]. Despite the significant increase in cervical cancer screening and the rising number of biopsies processed between 2020 and 2022, maintaining this TAT is noteworthy. This mirrors the laboratory’s effective management of its processes over time to ensure timely diagnoses despite the high number of tissue biopsies reported between 2020 and 2022. Ensuring a 14-day TAT helps improve cervical cancer outcomes by facilitating earlier intervention, reducing patient anxiety, and contributing to better public health outcomes [41]. 

However, the manual equipment and frequent malfunctions of automated equipment likely posed obstacles to efficiency, especially given the increased workload from cervical cancer biopsies. This reflects the broader challenge faced by many low- and middle-income countries (LMICs), where equipment shortages hinder the ability to provide timely and accurate diagnoses, exacerbating the burden of cervical cancer. As reported by Thomas et al. [13], adequate pathological resources are vital in ensuring early detection of cervical cancer in the curable stage. However, according to our study, Kenya, like many low- and middle-income countries (LMICs), faces a significant scarcity of diagnostic pathology facilities and staff. [13] This shortage exacerbates the cervical cancer burden, as delayed diagnoses result in advanced-stage presentations, reducing the likelihood of successful treatment and survival. Additionally, staff shortages often lead to burnout and diminished quality of service, which further impacts the timely diagnosis of cervical cancer cases [5]. These shortcomings highlight the persistent disparities in histopathology equipment inventories between LMICs and HICs.

### Strengths and limitations of the study

The strengths of this study include the use of mixed methods for retrospective data analysis to assess the histopathology laboratory inventory and the use of parallel interviews to corroborate the information for robustness. Using five years of data provided a holistic understanding of the evolution of laboratory capacity, offering a nuanced and comprehensive understanding of development over time. One limitation of this study is the reliance on a single interview with laboratory staff, which may introduce interviewer bias; however, cross-referencing the pathology laboratory inventory records was essential for confirming the information and ensuring comprehensive data analysis. To enhance the robustness of future research, we recommend conducting longitudinal cohort studies that track patients over time [42]. This study did not specifically address the availability and regular supply of consumables and reagents necessary for routine histology work, including Hematoxylin and Eosin (H&E) staining. This aspect is crucial as it directly impacts the consistency and quality of diagnostic services, even in well-equipped laboratories. Future studies should consider assessing the availability of these critical resources as part of the overall evaluation of histopathology services.

## Conclusions and recommendations

Our study underscores the critical role of histopathology resources in diagnosing cervical cancer. It highlights the persistent challenges faced by MTRH in meeting the demand for cervical cancer screening and diagnosis. While the laboratory has seen an increase in cervical cancer biopsy samples processed across the years, the decline in personnel-to-patient ratios and reliance on malfunctioning or manual equipment constrain its capacity to deliver timely and accurate diagnoses. Despite these limitations, the lab maintained a commendable 14-day turnaround time, reflecting efforts to optimize processes under challenging circumstances.

These findings call for urgent intervention by the Kenyan Ministry of Health and other stakeholders to address workforce shortages, upgrade histology equipment, and integrate automation to improve diagnostic efficiency. A holistic approach to resource allocation is essential, emphasizing the need to strengthen infrastructure and expand training programs to increase personnel to meet the rising demand for of the population. By bridging these gaps, Kenya and similar LMICs can enhance their capacity to provide timely and effective cervical cancer diagnosis, ultimately improving treatment outcomes and reducing disease burden.

## Electronic Supplementary Material

Below is the link to the electronic supplementary material.

Supplementary Material 1

Supplementary Material 2

## Data Availability

The data is stored in a password-protected file at the Global Health Initiative offices at Brown University (Arnold Lab 91 Waterman Street, Room 205, Providence, RI 02912) and can be requested through Nelson Anangwe (email: nelsonanangwe@gmail.com), the corresponding author, or Eileen Caffrey Wright ((email: eileen_caffrey@brown.edu), the Program Manager.

## Abbreviations

AMPATH: Academic Model Providing Access to Healthcare
ASCCP: American Society for Colposcopy and Cervical Pathology
EQA: External Quality Assessment
HIC: High-Income Country
HIV: Human immunodeficiency virus
HPV: Human Papilomavirus
ISO: International Standards Organization
KNH: Kenyatta National Hospital
LMIC: Low- and Middle-Income Country
MTRH: Moi Teaching and Referral Hospital
NHSCSP: National Health Service Cervical Screening Program
SSA: Sub-Saharan Africa
TAT: Turnaround Time
WHO: World Health Organization
WISN: Workload Indicators of Staffing Need

## References

[1] Bhatla N, et al. Cancer of the cervix uteri: 2021 update. Int J Gynecol Obstet. 2021;155:28–44. 10.1002/ijgo.13865 PMC9298213 34669203

[2] Sung H, et al. Global cancer statistics 2020: GLOBOCAN estimates of incidence and mortality worldwide for 36 cancers in 185 countries. Cancer J Clin. 2021;71(3):209–49. 10.3322/caac.21660 33538338

[3] Hull R, et al. Cervical cancer in low and middle-income countries. Oncol Lett. 2020;20(3):2058–74. 32782524 10.3892/ol.2020.11754 PMC7400218

[4] Cohen PA, et al. Cervical cancer. Lancet. 2019;393(10167):169–82. 30638582 10.1016/S0140-6736(18)32470-X

[5] Khozaim K, et al. Successes and challenges of establishing a cervical cancer screening and treatment program in western Kenya. Int J Gynecol Obstet. 2014;124(1):12–8. 10.1016/j.ijgo.2013.06.035 24140218

[6] Bulten J, et al. European guidelines for quality assurance in cervical histopathology. Acta Oncol. 2011;50(5):611–20. 21314297 10.3109/0284186X.2011.555779

[7] Nelson AM, et al. Oncologic care and pathology resources in Africa: survey and recommendations. J Clin Oncol. 2016;34(1):20–6. 26578619 10.1200/JCO.2015.61.9767

[8] Rosser JI, et al. Barriers to cervical cancer screening in rural Kenya: perspectives from a provider survey. J Community Health. 2015;40:756–61. 25677728 10.1007/s10900-015-9996-1 PMC8162879

[9] Mungo C, et al. Scaling up cervical cancer prevention in Western Kenya: treatment access following a community-based HPV testing approach. Int J Gynaecol Obstet. 2021;152(1):60–7. 32347550 10.1002/ijgo.13171 PMC8906492

[10] Gravitt PE, et al. Effectiveness of VIA, Pap, and HPV DNA testing in a cervical cancer screening program in a peri-urban community in Andhra Pradesh, India. PLoS ONE. 2010;5(10):e13711. 21060889 10.1371/journal.pone.0013711 PMC2965656

[11] Castle PE, Einstein MH, Sahasrabuddhe VV. Cervical cancer prevention and control in women living with human immunodeficiency virus. Cancer J Clin. 2021;71(6):505–26. 10.3322/caac.21696 PMC10054840 34499351

[12] Adesina A, et al. Improvement of pathology in sub-saharan Africa. Lancet Oncol. 2013;14(4):e152–7. 23561746 10.1016/S1470-2045(12)70598-3

[13] Randall TC, Ghebre R. Challenges in prevention and care delivery for women with cervical cancer in sub-saharan Africa. Front Oncol. 2016;6:160. 27446806 10.3389/fonc.2016.00160 PMC4923066

[14] *The pathologist*. 2023.

[15] Metter DM, et al. Trends in the US and Canadian pathologist workforces from 2007 to 2017. JAMA Netw open. 2019;2(5):e194337–194337. 31150073 10.1001/jamanetworkopen.2019.4337 PMC6547243

[16] *The Royal College of Pathologists*. 2024.

[17] WHO. *World Health Organization report on Cancer*. 2020.

[18] Stelzle D, et al. Estimates of the global burden of cervical cancer associated with HIV. Lancet Glob Health. 2021;9(2):e161–9. 33212031 10.1016/S2214-109X(20)30459-9 PMC7815633

[19] Kessler TA. Cervical Cancer: Prevention and early detection. Semin Oncol Nurs. 2017;33(2):172–83. 28343836 10.1016/j.soncn.2017.02.005

[20] Kivuti-Bitok LW, et al. An exploration of opportunities and challenges facing cervical cancer managers in Kenya. BMC Res Notes. 2013;6:136. 23566436 10.1186/1756-0500-6-136 PMC3626574

[21] Rosser JI, et al. Barriers to Cervical Cancer Screening in Rural Kenya: perspectives from a Provider Survey. J Community Health. 2015;40(4):756–61. 25677728 10.1007/s10900-015-9996-1 PMC8162879

[22] Ng’ang’a A, et al. Predictors of cervical cancer screening among Kenyan women: results of a nested case-control study in a nationally representative survey. BMC Public Health. 2018;18(Suppl 3):1221. 30400916 10.1186/s12889-018-6054-9 PMC6219012

[23] ICO/IARC Information Centre on HPV and Cancer. *Kenya Human Papillomavirus and Related Cancers, Fact Sheet* 2021, The Catalan Institute of Oncology (ICO) and the International Agency for Research on Cancer (IARC): Spain.

[24] Mwenda V, et al. Cervical cancer programme, Kenya, 2011–2020: lessons to guide elimination as a public health problem. Ecancermedicalscience. 2022;16:1442. 36200015 10.3332/ecancer.2022.1442 PMC9470178

[25] Kivuti-Bitok LW, et al. An exploration of opportunities and challenges facing cervical cancer managers in Kenya. BMC Res Notes. 2013;6(1):1–10. 23566436 10.1186/1756-0500-6-136 PMC3626574

[26] Ngutu M, Nyamongo IK. Exploring the barriers to health care and psychosocial challenges in cervical cancer management in Kenya. Int J women’s health. 2015:791–8. 10.2147/IJWH.S88668 PMC4556289 26346001

[27] Odondi RN, Shitsinzi R, Emarah A. Clinical patterns and early outcomes of burn injuries in patients admitted at the Moi Teaching and Referral Hospital in Eldoret, Western Kenya. Heliyon. 2020;6(3):e03629. 32258478 10.1016/j.heliyon.2020.e03629 PMC7096742

[28] MTRH, *Moi Teaching and Referral Hospital*, in *Moi Teaching and Referral Hospital*. 2024.

[29] Jacobsen J, et al. Towards Long Term Development in Kenya: a policy analysis. Int J Econ Bus Manage Res. 2023;7(06, 2023):246–62.

[30] Organization WH. *Workload indicators of staffing need.* 2010.

[31] Pereira P. *ISO 15189: 2012 Medical laboratories-Requirements for quality and competence.* Westgard QC: Madison, WI, USA, 2020.

[32] Organization WH. *WHO good practices for pharmaceutical quality control laboratories.* WHO Technical Report Series, 2010. 957.

[33] Improvement N. Cytology improvement guide—achieving a 14 day turnaround time in cytology. Leicester, UK: NHS Improvement; 2009.

[34] Green J, Thorogood N. *Qualitative methods for health research.* 2018.

[35] Strauss A, Corbin J. *Basics of qualitative research techniques.* 1998.

[36] Charmaz K. Constructing grounded theory: a practical guide through qualitative analysis. sage; 2006.

[37] Abdulkareem FB, Odubanjo OM, Awolola AN. *Pathological services in sub-Saharan Africa, a barrier to effective cancer care.* Cancer in Sub-Saharan Africa: Current Practice and Future, 2017: pp. 53–64.

[38] Zardin G, Braithwaite L. *11 Automation in the histology.* Bancroft’s Theory and Practice of Histological Techniques E-Book, 2018: p. 139.

[39] Pantanowitz L, Hornish M, Goulart RA. The impact of digital imaging in the field of cytopathology. Cytojournal. 2009;6. 10.4103/1742-6413.48606 PMC2678829 19495408

[40] Wu ES, Jeronimo J, Feldman S. Barriers and challenges to treatment alternatives for early-stage cervical cancer in lower-resource settings. J Global Oncol. 2017;3(5):572–82. 10.1200/JGO.2016.007369 PMC5646895 29094097

[41] Mumba JM, et al. Cervical cancer diagnosis and treatment delays in the developing world: evidence from a hospital-based study in Zambia. Gynecologic Oncol Rep. 2021;37:100784. 10.1016/j.gore.2021.100784 PMC8165546 34095422

[42] Hulley SB, et al. Designing cross-sectional and cohort studies. Designing Clin Res. 2013;4:85–96.

