# Supplementary material for: Evaluation of pathology resources for cervical cancer detection between 2018 and 2022: a retrospective study at Moi Teaching and Referral Hospital, Western Kenya
Source: BMC Cancer. 2025 Feb 5;25:203. doi: 10.1186/s12885-025-13563-9 (PMC11796189; doi:10.1186/s12885-025-13563-9)
Supplement: Supplementary file 1 — Supplementary Material 1 [file 12885_2025_13563_MOESM1_ESM.pdf]

BROWN Pathology resources inventory checklist:			Date_____/_____/_______ __
**Note taker (Initials):**	**Facility name**:	**County/Country**:	
**Contact Info:**	**Laboratory Department:**		
**YEAR:**			

A) INFRASTRUCTURE

1. Inventory of essential diagnostic and pathology instruments:

i) Pathology Equipment	Time acquired (Month/year)	Time commissioned for use (Month/year)	Working Condition/Notes/Rating
a. Cytology equipment			
b. Automatic tissue processor (ATP)			
c. Embedding machines			
d. Microtome			
e. Centrifuges			
f. Immunohistochemistry (IHC) machine			

4. How often are quality control and evaluation (including servicing) done for the pathology equipment mentioned?
a) Monthly b) Quarterly c) Semi-annually d) Yearly e) Other ______________________________

5. Is there any pathology equipment currently non-functional or broken? (Please state reason for non-functionality and duration): ____
____

6. How long is the duration between the collection of a sample/biopsy and the release of histopathology results from the lab?
i) <one week ii) 1-2 weeks iii) 3-4 weeks iv) 1-3 months iv) >3 months

B) PERSONNEL:

1. How many pathology personnel are available at the histopathology lab: (List+role+certifications). (probe: How many technicians)

Specialization	Number	Role + Years of experience

Pathologists		
Lab Technologists		
Records persons		
Office Admins		
Lab assistants		

C) REFERRAL SYSTEMS

1. How frequently are cases from the MTRH histopathology lab referred to other institutions for further testing?
   1. Rarely 2. Sometimes 3. Most times

2. Which cases are mostly referred if any? (list)

Obervation/Comments:____

____

Form Filled By: ___________________________ **Assisting Provider #:** _________________

Should the laboratory possess fewer than the required minimum essential components, it might be deemed insufficient for providing adequate cervical cancer histopathology services.
