# Supplementary material for: Evaluation of pathology resources for cervical cancer detection between 2018 and 2022: a retrospective study at Moi Teaching and Referral Hospital, Western Kenya
Source: BMC Cancer. 2025 Feb 5;25:203. doi: 10.1186/s12885-025-13563-9 (PMC11796189; doi:10.1186/s12885-025-13563-9)
Supplement: Supplementary file 2 — Supplementary Material 2 [file 12885_2025_13563_MOESM2_ESM.pdf]

An ISO 9001:2015 Certified Hospital

# MOI TEACHING AND REFERRAL HOSPITAL

## HEALTH RECORDS AND INFORMATION SERVICES DEPARTMENT

**Outpatient and Inpatient attendances for the years 2018 - 2023**

| YEAR | OUTPATIENT | INPATIENT |
|---|---|---|
| 2018 | 285688 | 45348 |
| 2019 | 345838 | 47756 |
| 2020 | 358051 | 39348 |
| 2021 | 464754 | 42748 |
| 2022 | 483337 | 42228 |
| 2023 | 439843 | 53937 |

**Prepared by:**

**Jackline Ronoh**
Statistics & Research
Health Records and Information Services
*23.02.2024*

**Confirmed By:**

Richard Ole Kuyo

**MHRIS**
